# Hadrosauroid Dinosaurs from the Late Cretaceous of the Sultanate of Oman

**DOI:** 10.1371/journal.pone.0142692

**Published:** 2015-11-12

**Authors:** Eric Buffetaut, Axel-Frans Hartman, Mohammed Al-Kindi, Anne S. Schulp

**Affiliations:** 1 Laboratoire de Géologie de l’Ecole Normale Supérieure, CNRS (UMR 8538), Paris, France; 2 Petroleum Development Oman, Muscat, Sultanate of Oman; 3 Shell International Exploration and Production B.V, The Hague, the Netherlands; 4 Naturalis Biodiversity Center, Leiden, the Netherlands; 5 Natuurhistorisch Museum Maastricht, Maastricht, the Netherlands; 6 Faculty of Earth and Life Sciences, Amsterdam VU University, Amsterdam, the Netherlands; Institute of Vertebrate Paleontology and Paleoanthropology Chinese Academy of Sciences, CHINA

## Abstract

Fragmentary post-cranial remains (femora, tibia, vertebrae) of ornithischian dinosaurs from the Late Cretaceous of the Sultanate of Oman are described and referred to hadrosauroids. The specimens come from the Al-Khod Conglomerate, of latest Campanian to Maastrichtian age, in the north-eastern part of the country. Although the fragmentary condition of the fossils precludes a precise identification, various characters, including the shape of the fourth trochanter of the femur and the morphology of its distal end, support an attribution to hadrosauroids. With the possible exception of a possible phalanx from Angola, this group of ornithopod dinosaurs, which apparently originated in Laurasia, was hitherto unreported from the Afro-Arabian plate. From a paleobiogeographical point of view, the presence of hadrosauroids in Oman in all likelihood is a result of trans-Tethys dispersal from Asia or Europe, probably by way of islands in the Tethys shown on all recent paleogeographical maps of that area. Whether hadrosauroids were widespread on the Afro-Arabian landmass in the latest Cretaceous, or where restricted to the « Oman island » shown on some paleogeographical maps, remains to be determined.

## Introduction

Very few dinosaur fossils have been reported from the Arabian Peninsula [1]. From the Sultanate of Oman, the first mention of dinosaur remains was made by Nolan et al. in 1990 [2] based on an indeterminate ornithischian vertebra from the Al-Khod Conglomerate. A reconnaissance in the Al-Khod Conglomerate in 1997 by ASS and AFH resulted in the discovery of additional fossil vertebrates [3], and fieldwork by an expanded team in 1998 yielded further material, including dinosaur, turtle and crocodile remains [4,5]. From 2011 until 2014 the second author returned to the Al Khod area on a regular basis for additional prospecting, which resulted in the discovery of numerous new (though mostly fragmentary) dinosaur remains. Here we describe a hadrosauroid assemblage from Al-Khod, found during that period. These specimens represent the first evidence of Late Cretaceous hadrosauroids from the Afro-Arabian continent.

The specimens were collected in Al Khod, Muscat, Sultanate of Oman, with permission of the Royal Botanic Garden Al Khod, the Diwan of the Royal Court and with the full support of the Geological Society of Oman. For further detailed analyses, the samples were temporarily taken to Paris and returned to Oman in April 2014, with an export permit issued by the Ministry of Commerce and Industry. The specimens described are stored in the Oman Natural History Museum in Muscat (part of the Ministry of Cultural Heritage), Al Khuwair. The specimens are registered as ONHM3744-3748.

### Locality, geological setting and stratigraphy

Prospecting efforts mainly focused on the type area of the Al-Khod Conglomerate Formation [2] exposed some 3 km SE of the village of Al-Khod. The deltaic conglomerates of the Al Khod Formation overlie the Semail Ophiolite, consisting of Tethyan oceanic crust that was obducted during the middle to late Campanian [6]. Material eroded from these newly obducted proto-Oman Mountains was deposited in fluvial and deltaic setting at the foothills (close to the shore, as revealed by the presence of abundant marine molluscs in the same beds as the dinosaur remains) and comprise the Al-Khod Formation. The age of the formation is bracketed between the obduction of the ophiolite and the extinction of the dinosaurs, and as such estimated to be latest Campanian to Maastrichtian. The Al-Khod Formation is approximately 800m thick at its type locality; lithology in a number of outcrops was logged and sampled in detail in 1998, and described by Schulp et al. [3]. The unit consists of a series of clast-supported conglomerates interbedded with sandstone, silts and clays. Nolan et al. [2] considered the Al-Khod Conglomerate to have formed in a fan deltaic setting, which seems to be supported by the presence of high angle mega-ripples.

The material described in this contribution was found near the base of the Al-Khod Formation at 40 m (true stratigraphic thickness) above the Semail Ophiolite. The contact between the two units is abrupt due to the presence of a NW-SE trending boundary fault, with an azimuth of 130 degrees. The nearby presence of this fault system may have contributed to the heavily fractured nature of the material.

## Systematic Paleontology

There is little consensus about the detailed phylogeny of “hadrosaurs” *sensu lato*. A number of taxa considered as more advanced than iguanodontids but less so than highly derived forms such as hadosaurines and lambeosaurines are often loosely referred to as “basal hadrosauroids”. They include such taxa as *Telmatosaurus*, *Gilmoreosaurus* and *Yunganglong*, to cite but a few. The material from Oman described in the present paper poses nomenclatural problems because it only consists of isolated and incomplete postcranial elements. Because of this, or main purpose has been to determine, as far as possible, whether this material belongs to “hadrosaurs” or to more basal ornithopods such as iguanodontids, bearing in mind that the distinction may be difficult on the basis of fragmentary material. We have chosen to use the term “hadrosauroid”, as used, notably, by McDonald [7], to denote ornithopods more advanced than iguanodontids, without trying to reach a degree of nomenclatural precision that would not be warranted by the available fossil material.

### Description

Among the numerous skeletal remains recovered from the Al-Khod Conglomerate, many are too fragmentary to be identified beyond “dinosaur”. No complete bones have been found and most specimens have undergone some abrasion. Limb bones, in particular, are represented only by fragments. Several of them, however, show distinctive features, allowing referral to hadrosauroid dinosaurs.

#### Femora

**Fig 1:**
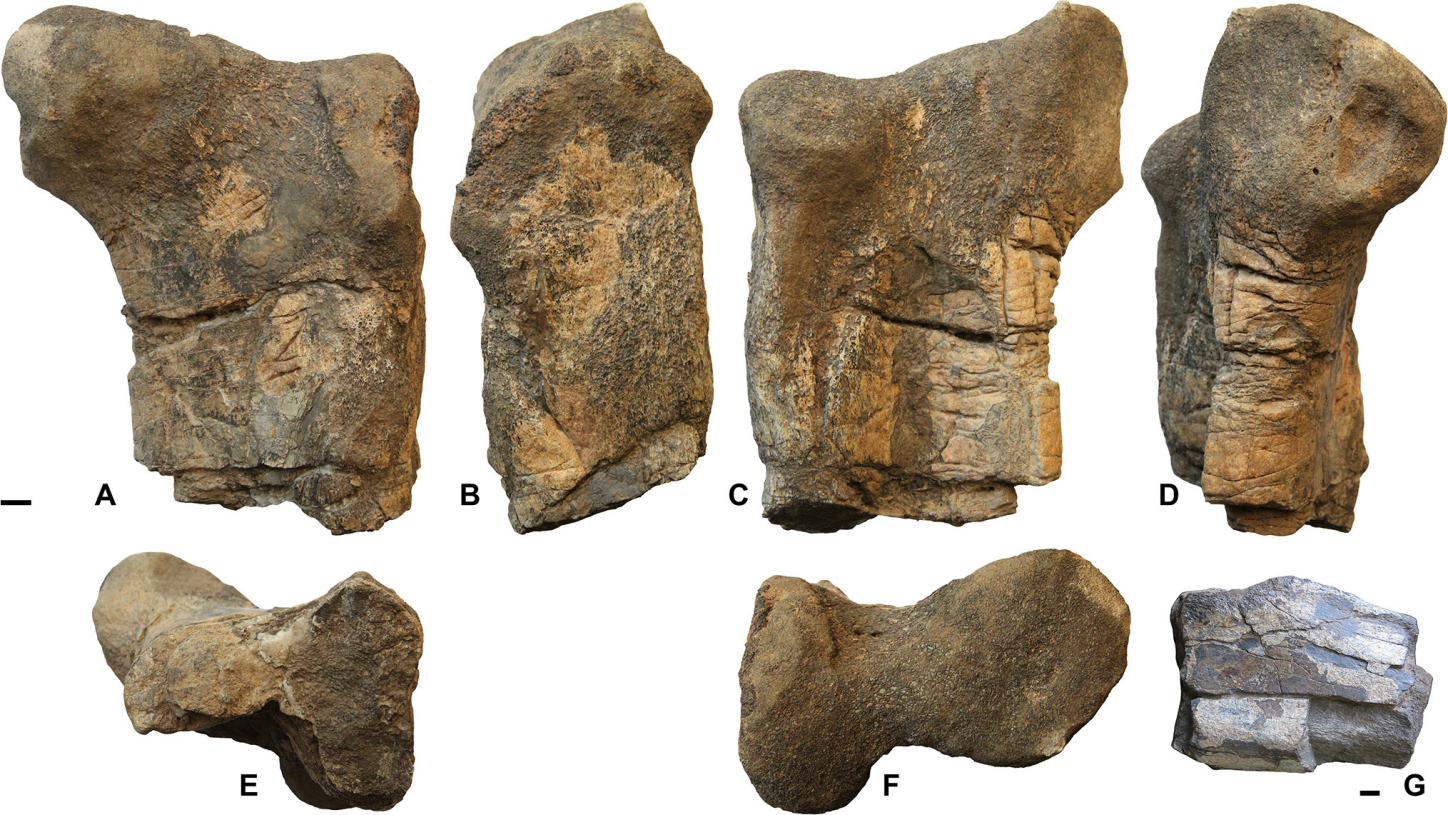
Hadrosauroid femora from the Al-Khod Conglomerate, Sultanate of Oman. Proximal end of left femur ONHM 3748 in anterior (A), lateral (B), posterior (C), medial (D), distal (E) and proximal (F) view, and shaft fragment ONHM 3746 [G] with fourth trochanter preserved. Scale bars equal 1 cm.

Several femoral fragments were recovered, including one consisting of the middle part of the shaft, with the fourth trochanter, a proximal end and three distal ends (Fig 1). The proximal end of a left femur is fairly well preserved, although it has suffered some abrasion. It shows a well developed hemispherical head at right angles to the shaft. There is a well defined, proximally prominent great trochanter along the lateral margin of the specimen. The lesser trochanter is poorly individualized, there is no apparent cleft between it and the greater trochanter and, despite the imperfect preservation of this area, it seems that the lesser and greater trochanters were confluent, as, for instance, in *Hadrosaurus* [8]. As noted by Brett-Surman and Wagner [9, p. 156], in hadrosaurs “the lesser trochanter displays considerable variation in size, orientation and degree of fusion to the greater trochanter”, but this individual variation cannot be used to distinguish species. However, in more basal ornithopods, including rhabdodontids [10] and iguanodontids [11–13], the lesser trochanter is clearly separated from the greater trochanter by a well-marked cleft. Therefore, the condition seen on the specimen from Oman suggests hadrosauroid affinities rather than more basal iguanodontian ones.

The shaft fragment is significant because it shows a nearly complete, although rather abraded, fourth trochanter. The trochanter is a low, thick S-shaped ridge along the mediocaudal edge of the shaft. Despite some abrasion, it is sufficiently well preserved to show that it does not end in a sharp point and is not pendant, since its distal margin shows no sign of a distally facing concavity. The shape of the fourth trochanter is therefore different from the “pendant” condition seen in relatively basal ornithopods, including rhabdodontids [10] and iguanodontids [11–14] and more similar to what is seen in hadrosauroids [7]. Brett-Surman and Wagner [9] distinguish the triangular outline of the fourth trochanter seen in large hadrosaurs from the low, rounded shape seen in smaller forms. This would be in agreement with the relatively small size of the specimen from Oman. Prieto-Márquez [15] considers a smooth and arcuate profile of the caudoventral margin of the fourth trochanter to be a derived feature in hadrosauroids. Likewise, McDonald [7] lists a fourth trochanter forming a curved, laterally compressed eminence, as the most derived condition in ornithopods. The morphology of the fourth trochanter thus supports referral of the Omani material to hadrosauroids.

**Fig 2:**
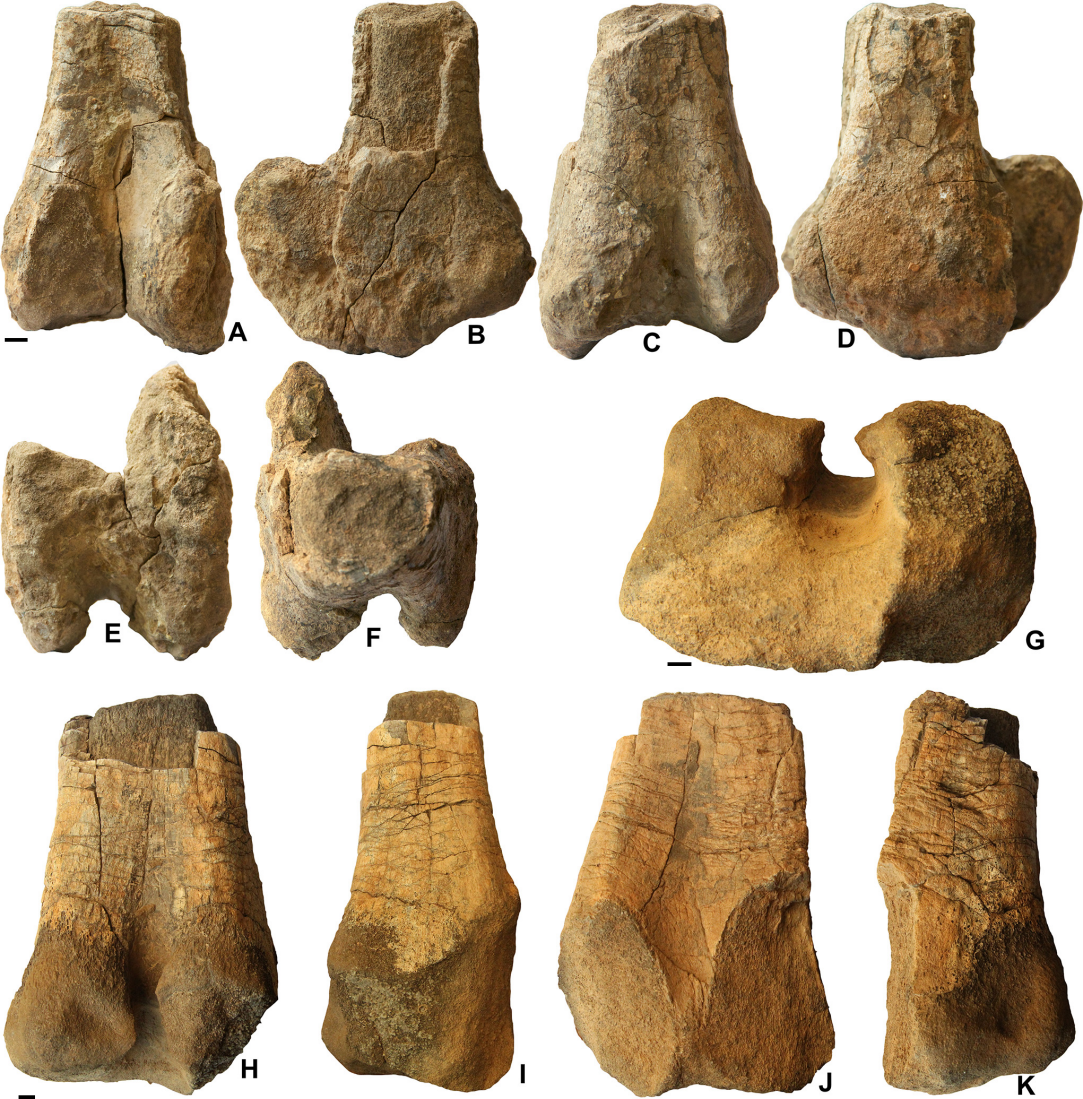
Hadrosauroid femora from the Al-Khod Conglomerate, Sultanate of Oman. Distal end of left femur ONHM 3744 in posterior (A), medial (B), anterior (C), lateral (D), distal (E) and proximal (F) view. Distal end of left femur ONHM 3745 in distal (G), posterior (H), medial (I), anterior (J) and lateral (K) view. Scale bars equal 1 cm.

The distal end of the femur (Fig 2) is represented by two specimens that show the same morphology. The shaft is flattened craniocaudally. The condyles are strongly developed. The medial condyle is thicker than the lateral one. Both protrude considerably caudally, with a somewhat recurved proximal margin, and are separated by a deep and wide groove. Cranially, they also protrude markedly and are separated by a deep and narrow cleft for tendons that reached the proximal end of the tibia (the anterior tendinal canal of Brett-Surman [9]). Although their cranial ends are close to each other, the condyles do not completely close over the cleft. The cranial protrusion of the condyles is not present in rhabdodontids, in which the space between the condyles on the cranial side of the bone is broad and shallow [10] but it occurs, to various degrees, in more advanced ornithopods, including iguanodontids [11–13] and hadrosaurs [8,16], in which the anterior tendinal canal is deep and narrow. In some hadrosauroids the cranial ends of the condyles meet each other, thus completely enclosing the anterior tendinal canal in bone (the so-called “extensor tunnel”). Whether this is size-related seems unclear (see discussion in [9]). However that may be, the specimens from Oman do not show such a closure of the canal, but the anterior protrusion is notable, apparently more marked than in most iguanodontids. Therefore, the morphology of the distal end of the femur seems more suggestive of hadrosauroid than of iguanodontid affinities. Similarities with various basal hadrosauroids, such as *Yunganglong* [17] and *Gilmoreosaurus* [18], are worth noting, notably because there is no extensor tunnel. However, the phylogenetic significance of this character is far from clear, as it is present in some basal taxa such as *Telmatosaurus* [19] and not fully developed in derived forms such as *Edmontosaurus* [20].In addition, it is subject to variability. More important than the presence or absence of an extensor tunnel may be the cranial protrusion and expansion of the condyles, which is generally more accentuated in hadrosauroids than in iguanodontids. In this regard, the condition observed in the Omani specimens corresponds to derived character state 3 listed by McDonald [7] and is in agreement with an attribution to hadrosauroids, although admittedly some advanced iguanodontids come close to that morphology.

#### Tibia

**Fig 3:**
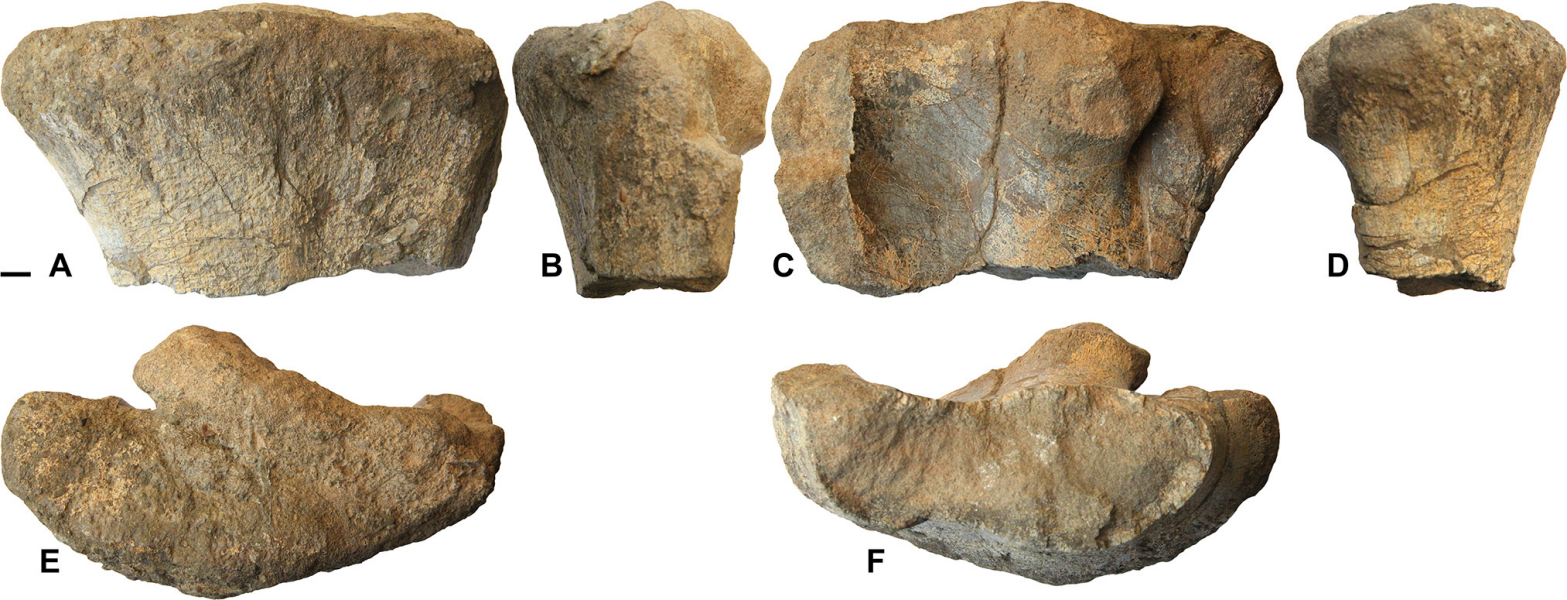
Hadrosauroid tibia from the Al-Khod Conglomerate, Sultanate of Oman. Proximal end of left tibia ONHM 3747 in medial (A), anterior (B), lateral (C), posterior (D), proximal (E) and distal (F) view. Scale bar equals 1 cm.

A well preserved proximal end of a left tibia (Fig 3) shows a well-developed cnemial crest that curves markedly laterally. The tibial condyles are separated by a deep and narrow furrow. The medial condyle is larger than the lateral one and extends farther caudally. The general morphology of the articular head of the tibia indicates an advanced iguanodontian. More basal ornithopods, including rhabodontids [10], show a less pronounced cnemial crest and less well individualized condyles. However, the fragmentary nature of the Al-Khod specimen makes it difficult to decide whether it is more hadrosaur-like than iguanodontid-like. The only character from the tibia used in the phylogenetic analysis in [15] (cnemial crest extending down the shaft) cannot be observed on the specimen from Oman.

#### Vertebrae

Most vertebral elements recovered so far are isolated centra. Their morphology is compatible with referral to iguanodontians but little more can be said about them.

## Results and Discussion

The material described here clearly belongs to ornithopod dinosaurs, as shown especially by the morphology of the femora and tibiae. A more accurate placement among ornithopods is possible mainly on the basis of femoral morphology. As noted above, all the characters observable on the femur and tibia indicate an advanced iguanodontian and are not compatible with referral to rhabdodontids.

The main question is thus to determine whether the bones from Oman belong to a hadrosauroid or to a less derived iguanodontian. This is no easy task because of the fragmentary nature of the available material and of the many similarities in the limb elements among iguanodontians as a whole. Nevertheless, as mentioned above several characters of the femur appear to be more reminiscent of hadrosauroids than of iguanodontids. In particular, the shape of the fourth trochanter seems to be distinctive. The fusion of the lesser trochanter with the greater trochanter may be an additional hadrosauroid character. The morphology of the distal end of the femur may be less characteristic, but the strong cranial protrusion and expansion of the distal condyles is also strongly reminiscent of hadrosauroids.

It is therefore concluded that the ornithopod material described in the present contribution can be referred to hadrosauroids. A more precise systematic placement cannot be attempted because of the incomplete nature of the material.

### Paleobiogeographical significance

Although various dinosaur remains have been reported from the Mesozoic of the Arabian Peninsula and the Levant (reviewed in [1]), including Oman [3,4], no hadrosauroids had so far been reported from that area. More generally, hadrosauroids were previously unknown from the Afro-Arabian plate [15] (although a possible hadrosauroid pedal phalanx from the Maastrichtian of Angola [21: Fig 17] is worth mentioning). The group has an essentially Laurasian distribution, with the exception of South American forms that dispersed from North America no later than the Late Campanian [15]. The biogeographical history of derived hadrosauroids (Hadrosauridae) is generally considered as having take place primarily in North America and Asia [22], but that of more basal hadrosauroids seems to be less well understood, possibly because the phylogenetic relationships of these forms is less well understood. However, basal hadrosauroids are mainly known from Asia and to some extent North America, with interesting records from Europe. The presence of hadrosauroids in Oman, i.e., the north-eastern part of the Afro-Arabian plate, in the Maastrichtian is therefore unexpected. The recently described dinosaurs from Saudi Arabia [1], of Campanian-Maastrichtian age, are titanosaurid sauropods and abelisaurid theropods and therefore belong to groups that were widespread in Afro-Arabia during the Late Cretaceous. The hadrosauroids from Oman are more difficult to interpret from a paleobiogeographical point of view. They can hardly be considered to be the result of local evolution on the Afro-Arabian plate, because it is generally accepted that the evolution of hadrosaurs mainly took place in Laurasia [15]. Although iguanodontians are known from the Early Cretaceous of Africa, the best-known form being *Ouranosaurus* from Niger [11], there is no evidence whatsoever that more derived forms such as hadrosaurs evolved in that part of the world. Admittedly, the Late Cretaceous dinosaur record from Afro-Arabia remains scanty, especially for the later part of that period, but what was known so far indicated the prevalence of “Gondwanan” forms such as titanosaurs and abelisaurids, which are known, for instance, from the Maastrichtian phosphates of Morocco [23,24], as well as from Saudi Arabia [1].

If a local origin and evolution is dismissed, the presence of hadrosauroids in the Maastrichtian of Oman needs to be explained by dispersal. Most paleogeographical reconstructions for the Maastrichtian (e.g.,[15,25,26]) do not show much if any degree of close contact between the Afro-Arabian continental mass and Eurasia, the Tethys forming a marine barrier between them. In addition, the north-eastern part of Oman where the hadrosauroids come from is reconstructed as an island separated from the Afro-Arabian mainland. This suggests that transoceanic dispersal must have been involved to bring hadrosauroids to the Oman region in the Late Cretaceous. From the point of view of geographical proximity, the most likely source for the Omani hadrosauroids is Asia, where that group of dinosaurs was widespread and diverse in the Late Cretaceous [15]. Hadrosauroids are also known from the Late Cretaceous of Europe, where they become abundant especially in the Maastrichtian, but the distance between Europe and Oman is clearly greater than that between Oman and Asia. However, a point worth considering in this context is that paleogeographical maps for the Maastichtian generally show strings of islands extending in a general NW-SE direction in the Tethys between Eurasia and Arabia-Africa (e.g., [15: p. 511, 25, 26]). These may have provided “stepping-stones” for hadrosauroid dispersal between Eurasia and the northeastern Arabian archipelago. The possible role of carbonate platforms should also not be underestimated. Examples from the Mediterranean area show that dinosaur populations, including hadrosauroids [27,28, 29] flourished on such platforms in the western part of the Tethys during emergent phases in the Cretaceous. The chain of islands and carbonate platforms stretching from the western part of the Tethys to the north-eastern corner of the Afro-Arabian plate in the latest Cretaceous may have made it possible for hadrosauroids to disperse from southern Europe to Oman, especially during episodes of marine regression, without implying direct dispersal from Asia.

**Fig 4:**
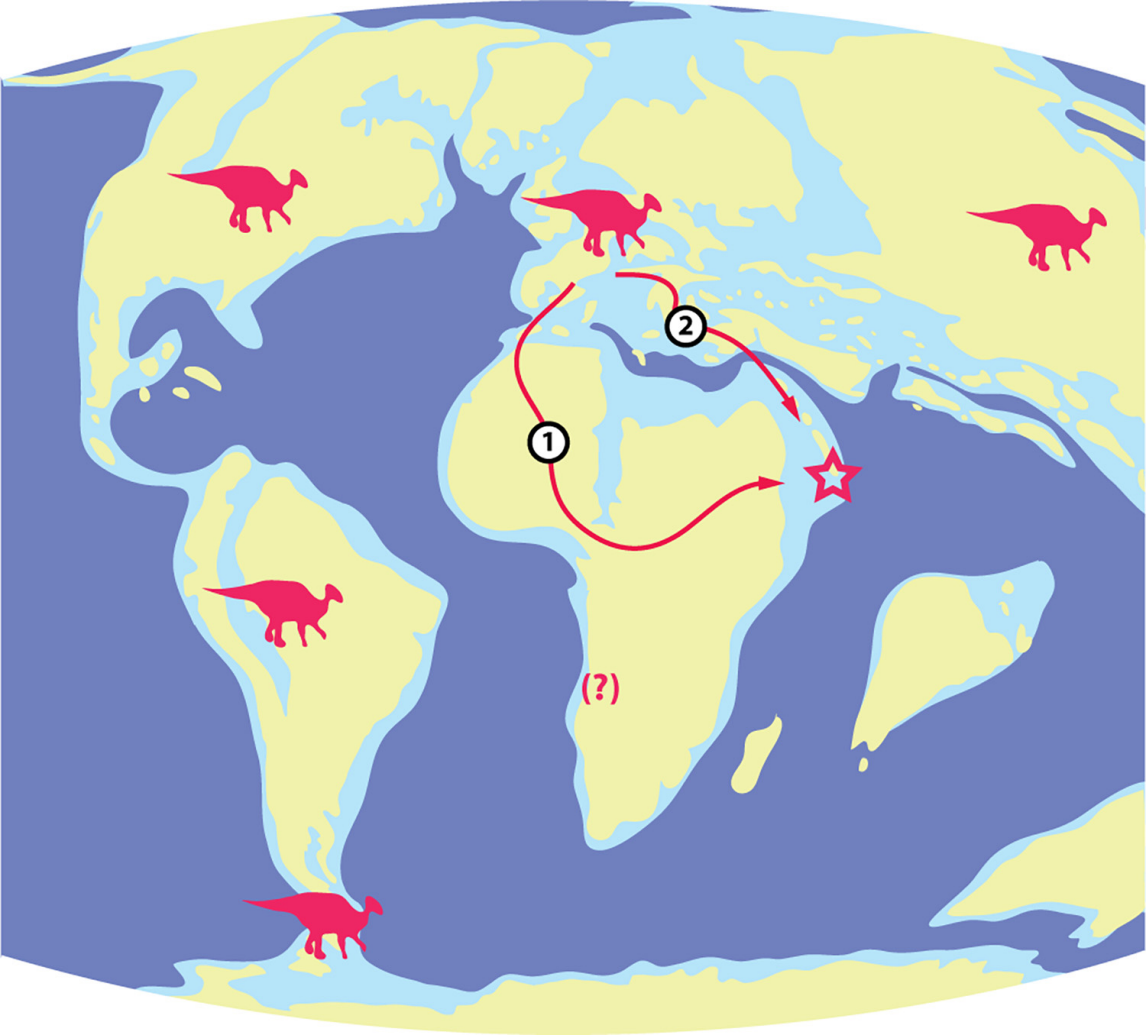
Suggested dispersal routes for hadrosauroids from Eurasia to the Late Cretaceous North-East Arabian archipelago of the Sultanate of Oman. Paleogeographic map simplified after [14].

A better reconstruction of the route followed by hadrosauroids when dispersing from Eurasia to Oman will depend on improving our current understanding of the paleogeography in the region, and the discovery of more complete material–which in turn may allow a more precise establishment of the phylogenetic affinities of the Omani hadrosauroids and thus help to pinpoint a more precise area of origin in Asia or Europe. Beyond that, the question remains whether hadrosauroids had really become part of the Afro-Arabian dinosaur fauna of the Maastrichtian, or whether they were restricted to the “Oman Island” shown by most paleogeographical reconstructions [15,19]. As noted above, there is currently no evidence of hadrosauroids from other parts of Afro-Arabia in the Late Cretaceous except for the isolated possible record from Angola [21]. Even the (admittedly small) dinosaur assemblage from Saudi Arabia does not contain hadrosauroids [1]. The possibility that the Maastrichtian dinosaurs from Oman may represent an insular assemblage cannot be ruled out. This could explain the relatively small dimensions of most of the hadrosauroid specimens. On the basis of what is currently known, the dinosaurs from Oman include sauropods, a theropod, a possible rhabdodontid [4] and the hadrosauroids described in the present paper. If the occurrence of a rhabdodontid is confirmed, this assemblage would be similar to that known from the Transylvanian Island in the Late Cretaceous. However, the presence of a rhabdodontid is, at this point, based on a single incomplete dorsal vertebra and must therefore be considered with caution, especially in view of the occurrence of abundant representatives of another group of ornithopods, viz. hadrosauroids. A proper assessment of the dinosaur assemblage from the Maastrichtian of Oman will clearly depend on the discovery of additional material. However, the occurrence of hadrosauroids in Oman can be considered to be well established and constitutes an unexpected addition to the geographical distribution of that group of dinosaurs (Fig 4).

## Abbreviations

ONHM: Oman Natural History Museum
Muscat: Sultanate of Oman

## References

[1] Kear BP, Rich TH, Vickers-Rich P, Ali MA, Al-Mufarreh YA, Matari AH, et al First Dinosaurs from Saudi Arabia. PLoS ONE. 2013;8(12): e84041 10.1371/journal.pone.0084041 24386326 PMC3873419

[2] Nolan SC, Skelton PW, Clissold BP, Smewing JD. Maastrichtian to early Tertiary stratigraphy and palaeogeography of the central and northern Oman Mountains. In: Robertson AHF, Searle MP, Ries AC, editors. The geology and tectonics of the Oman region. Geol Soc Lond. 1990; Sp Pub 49: 495–519.

[3] Schulp AS, Hanna SS, Hartman AF, Jagt JWM. A Late Cretaceous theropod caudal vertebra from the Sultanate of Oman. Cret Res. 2000;21: 851–856.

[4] Schulp AS, O’Connor PM, Weishampel DB, Al-Sayigh AR, Al-Harthy A, Jagt JWM, et al Ornithopod and sauropod dinosaur remains from the Maastrichtian Al-Khod Conglomerate, Sultanate of Oman. Sultan Qaboos Univ J Sc. 2008;13: 27–32.

[5] Buscalioni AD, Schulp AS, Jagt JWM, Hanna SS, Hartman AF. Late Cretaceous neosuchian crocodiles from the Sultanate of Oman. Cret Res. 2004;25: 267–275.

[6] Glennie KW, Boeuf MGA, Hughes Clarke MW, Moody-Stuart M, Pilaar WFH, Reinhardt BM. Geology of the Oman Mountains. Verh Kon Ned Geol Mijnb Gen. 1974;31: 1–423.

[7] McDonald AT. Phylogeny of Basal Iguanodonts (Dinosauria: Ornithischia): An Update. PLoS ONE 2012;7(5): e36745 10.1371/journal.pone.0036745 22629328 PMC3358318

[8] Leidy J. Cretaceous reptiles of the United States. Smithsonian Contr Knowl. 1865;192: 1–135.

[9] Brett-Surman MK, Wagner JR. Discussion of Character Analysis of the Appendicular Anatomy in Campanian and Maastrichtian North American Hadrosaurids—Variation and Ontogeny In: Carpenter K, editor. Horns and Beaks—Ceratopsian and Ornithopod Dinosaurs. Bloomington: Indiana University Press; 2006 pp. 135–169.

[10] Weishampel DB, Jianu C-M, Csiki Z, Norman DB. Osteology and phylogeny of *Zalmoxes* (n.g.), an unusual euornithopod dinosaur from the latest Cretaceous of Romania. J Syst Pal. 2003;1: 65–123.

[11] Taquet P. Géologie et paléontologie du gisement de Gadoufaoua (Aptien du Niger). Cah Paléont C.N.R.S. 1976;17: 1–191.

[12] Norman DB. On the ornithischian dinosaur *Iguanodon bernissartensis* of Bernissart (Belgium). Mém Inst R Sc Nat Belgique. 1980;178: 1–105.

[13] Norman DB. On the anatomy of *Iguanodon atherfieldensis* (Ornithischia: Ornithopoda). Bull Inst R Sc Nat Belgique, Sc Terre. 1986;56: 281–372.

[14] Dollo L. Note sur les restes de dinosauriens recontrés dans le Crétacé Supérieur de la Belgique. Bull Mus R Hist Nat Belgique. 1883;2: 205–221.

[15] Prieto-Márquez A. Global phylogeny of Hadrosauridae (Dinosauria: Ornithopoda) using parsimony and Bayesian methods. Zool J Linn Soc. 2010;159: 435–502.

[16] Lull RS, Wright NE. Hadrosaurian dinosaurs of North America. Geol Soc Am Special Papers. 1942;40: 1–242.

[17] Wang R, You H, Xu S, Wang S, Yi J, Xie L, et al A New hadrosauroid dinosaur from the early Late Cretaceous of Shanxi Province, China. PLoS ONE 2013;8(10): e77058 10.1371/journal.pone.0077058 24204734 PMC3800054

[18] Prieto-Márquez A, Norrell MA. Anatomy and Relationships of *Gilmoreosaurus mongoliensis* (Dinosauria: Hadrosauroidea) from the Late Cretaceous of Central Asia. Am Mus Nov. 2010;3694: 1–49.

[19] Weishampel DB, Norman DB, Grigorescu D. *Telmatosaurus transsylvanicus* from the Late Cretaceous of Romania: the most basal hadrosaurid dinosaur. Palaeontology. 1993;36: 361–385.

[20] Campione NE. Postcranial anatomy of *Edmontosaurus regalis* (Hadrosauridae) from the Horseshoe Canyon Formation, Alberta, Canada In: Eberth DA, Evans DC, editors. Hadrosaurs. Bloomington: Indiana University Press; 2014 pp. 208–244.

[21] Mateus O, Polcyn MJ, Jacobs LL, Araújo R, Schulp AS, Marinheiro J, et al Cretaceous amniotes from Angola: dinosaurs, pterosaurs, mosasaurs, plesiosaurs, and turtles. Actas V Jornadas Int Pal de Dinosaurios y su Entorno. 2012;5: 71–105.

[22] Prieto-Márquez A. Global historical biogeography of hadrosaurid dinosaurs. Zool J Linn Soc. 2010;159: 503–525.

[23] Pereda Suberbiola X, Bardet N, Iarochène M, Bouya B, Amaghzaz M. The first record of a sauropod dinosaur from the Late Cretaceous phosphates of Morocco. J Afr Earth Sci. 2004;40: 81–88.

[24] Buffetaut E, Escuillié F, Pohl B. First theropod dinosaur from the Maastrichtian phosphates of Morocco. Kaupia. 2005;14: 3–8.

[25] Camoin G, Bellion Y, Benkhelil J, Cornée JJ, Dercourt J, Guiraud R, et al Late Maastrichtian palaeoenvironments (69.5–65 Ma) In: Dercourt J, Ricou LE, Vrielynck B, editors. Atlas Tethys Palaeoenvironmental Maps. Rueil-Malmaison: BEICIP-FRANLAB; 1993.

[26] Vrielynck B, Bouysse P. Le visage changeant de la Terre; l’éclatement de la Pangée et la mobilité des continents au cours des derniers 250 millions d'années Paris: Comm Carte Géol Monde; 2001.

[27] Bosellini A. Dinosaurs “re-write” the geodynamics of the eastern Mediterranean and the paleogeography of the Apulia Platform. Earth Sci Rev. 2002;59: 211–234.

[28] Debeljak I, Košir A, Buffetaut E, Otoničar B. The Late Cretaceous dinosaurs and crocodiles of Kozina (SW Slovenia): a preliminary study. Mem Soc Geol It. 2002;57: 193–201.

[29] Dalla Vecchia FM. *Tethyshadros insularis*, a new hadrosauroid dinosaur (Ornithischia) from the Upper Cretaceous of Italy. Journal of Vertebrate Paleontology. 2009;29: 1100–1116.

